# Public attitudes about the support for the establishment of smoke-free environment

**DOI:** 10.3389/fpubh.2025.1646224

**Published:** 2025-12-16

**Authors:** Jing Kong, Yuanyuan Rong, Jingjing Yang, Tingting Niu, Mingjun Xu, Xinxin Liu, Tingyu Duan, Liang Chen

**Affiliations:** 1 The Key Laboratory of Cardiovascular Remodeling and Function Research, Chinese Ministry of Education, Chinese National Health Commission and Chinese Academy of Medical Sciences, The State and Shandong Province Joint Key Laboratory of Translational Cardiovascular Medicine, Department of Cardiology, Qilu Hospital of Shandong University, Jinan, Shandong, China; 2 Department of Cardiology, Qingdao Municipal Hospital; Qingdao Hospital, University of Health and Rehabilitation Sciences (Qingdao Municipal Hospital), Qingdao, Shandong, China; 3 Department of Emergency, Qilu Hospital of Shandong University, Jinan, Shandong, China; 4 Jinan Vocational College of Nursing, Jinan, Shandong, China; 5 Department of Ultrasound, Qilu Hospital of Shandong University, Jinan, Shandong, China; 6 Hebei Institute of Communications, School of Journalism and Communication, Shijiazhuang, China

**Keywords:** smoke-free environment, public support, influencing factors, China, FHS-SF

## Abstract

This study investigated factors influencing public support for smoke-free environments in China. A cross-sectional survey was conducted among 11,031 adults (45.6% male, 54.4% female) between July and September 2021. The mean support score was 78.75 (SD = 26.996), with the highest scores observed in Central China (e.g., Shanxi, Henan). Multiple regression analysis revealed that being female (*β* = 5.505, 95% CI: 4.409–6.601), having an undergraduate education or above (*β* = 4.110, 95% CI: [2.475–5.746]), and having children (*β* = 1.554, 95% CI: 0.276–2.833) were associated with stronger support. Conversely, being married (*β* = −3.375, 95% CI: −4.849 to −1.900), frequent drinking (e.g., weekly: *β* = −5.275, 95% CI: −6.851 to −3.699), and higher levels of smoking dependence (e.g., severe: *β* = −21.968, 95% CI: −27.671 to −16.265) were associated with lower support. Psychosocial factors were also significant. Greater family health (FHS-SF: *β* = 0.645, 95% CI: 0.543–0.746) and social support (PSSS: *β* = 0.142, 95% CI: 0.092–0.193) predicted stronger support, while a higher BFI-10 total score (BFI-10: *β* = −0.559, 95% CI: −0.853 to −0.266) and unhealthy eating habits (EBS-SF: *β* = −0.328, 95% CI: −0.444 to −0.212) predicted weaker support. Subgroup analyses among smokers and ex-smokers confirmed the negative associations of drinking, tobacco dependence, and poor diet with support levels. These findings underscore the multifaceted nature of public support for smoke-free policies in China. Interventions that strengthen family and social support systems, alongside targeted strategies for key demographics, may be particularly effective in bolstering public endorsement.

## 1 Introduction

Tobacco use remains a critical global public health challenge, with over 1 billion consumers worldwide (1, 2). China bears a particularly heavy burden, having the world’s largest population of smokers. The health consequences are severe and pervasive, damaging nearly every organ system and causing a spectrum of diseases from cancer to respiratory and cardiovascular conditions (Supplementary Figure 1). It is a leading cause of preventable death globally. In China alone, tobacco use claims over 1 million lives annually, a figure projected to rise without effective intervention (3, 4). In response, the World Health Organization (WHO) has prioritized tobacco control, advocating for evidence-based policies through the WHO Framework Convention on Tobacco Control (FCTC) (5). Aligning with the FCTC, China has taken legislative steps to establish smoke-free environments. However, the implementation and effectiveness of these policies face significant challenges, and their ultimate success is critically dependent on strong public support (6, 7). A critical, yet often overlooked, determinant for overcoming these implementation challenges is strong public support. While previous research, such as the ITC China Survey, has identified basic sociodemographic correlates of support, significant evidence gaps persist. Existing studies often lack a comprehensive framework that integrates the full spectrum of influences, from personal characteristics and health behaviors to underlying psychosocial mechanisms and broader societal factors. Furthermore, it remains unclear whether these correlates are consistent within key subgroups, such as current and former smokers, whose support is paramount for policy efficacy. To address these gaps, this study employs a socio-ecological framework (8) to systematically investigate the multifaceted factors shaping public support for smoke-free environments in China. This model allows us to examine factors across multiple levels: (1) intrapersonal (e.g., demographics, personality); (2) individual behaviors (e.g., smoking, drinking, diet); (3) interpersonal (e.g., social support, family health); (4) societal (e.g., education, occupation); and (5) smoking-specific factors (e.g., nicotine dependence). This study therefore has three primary objectives: first, to assess the current level of public support for smoke-free environments in China; second, to identify and quantify the influence of factors across the socio-ecological spectrum associated with this support; and third, to examine the persistence of these associations within the key subgroup of smokers and ex-smokers. By employing this comprehensive approach, our research aims to provide a nuanced evidence base to inform targeted communication strategies and policy interventions, thereby advancing the establishment of effective and sustainable smoke-free environments in China.

## 2 Materials and methods

### 2.1 Study design and sampling

This population-based cross-sectional study was conducted in Mainland China from July 10 to September 15, 2021. A multi-stage, hybrid sampling strategy was employed to recruit participants from 120 cities, aiming to capture geographic and socioeconomic diversity.

Stage 1: City Selection. A stratified hybrid sampling strategy was adopted. First, we purposively included all 32 provincial capitals and municipalities directly under the central government (e.g., Beijing, Shanghai) to ensure coverage of major administrative and population centers. Subsequently, for each province/autonomous region, we randomly selected 2 to 6 non-capital prefecture-level cities from a complete administrative division list using a random number table, resulting in a total of 120 cities.

Stage 2: Investigator Recruitment and Training. In each selected city, local investigators or investigation teams (with ≤10 members per team) were recruited through open channels, constituting a convenience sample. All investigators underwent standardized online training to ensure a consistent understanding of the survey protocol, questionnaire content, and ethical guidelines.

Stage 3: Participant Recruitment. Due to the absence of a comprehensive sampling frame for residents, a non-probability sampling method was employed at the individual level. Investigators distributed the online questionnaire (hosted on the Wenjuanxing platform) primarily through their personal and social networks (e.g., community and professional WeChat groups). Each independent investigator was tasked with collecting 30–90 valid questionnaires, while each team was responsible for 100–200. This approach combined elements of convenience and snowball sampling, with no randomized selection of individuals.

The selected cities collectively represent the major geographic (Eastern, Central, Western regions) and socioeconomic strata of mainland China, capturing a diverse range of occupational sectors and community types (see in the Supplementary material).

### 2.2 Participants and data source

The data were derived from the 2021 China Family Health Index Survey (CFHI-2021). The survey was administered online via the Wenjuanxing platform. Investigators distributed questionnaire links one-on-one to residents in their networks. Participants provided electronic informed consent before completing the questionnaire, and investigators recorded the questionnaire numbers.

Inclusion Criteria: ① Chinese nationality; ② Permanent resident in China (annual time away from residence ≤ 1 month); ③ Voluntary participation with informed consent; ④ Ability to complete the questionnaire independently or with investigator assistance; ⑤ Capacity to comprehend the questionnaire items.

Exclusion Criteria: ① Delirium or severe mental disorders; ② Concurrent participation in similar research projects; ③ Unwillingness to cooperate.

A participant flowchart is provided in Supplementary Figure 3.

### 2.3 Measures and reliability

The primary outcome was the level of support for establishing smoke-free environments, measured by a self-reported support score. The questionnaire also collected comprehensive data on: Sociodemographic characteristics: sex, age, education, occupation, marital status, etc. (see Supplementary Table 1 for categories). Standardized Scales: 10-item Big Five Inventory (BFI-10) for personality (9), Perceived Social Support Scale (PSSS), Family Health Scale-Short Form (FHS-SF), Eating Behavior Scale-Short Form (EBS-SF), Fagerström Test for Nicotine Dependence (FTND, For never-smokers and former smokers who had quit, FTND scores were set to zero), EQ-5D-5L for quality of life. For the primary analysis, a total BFI-10 score was used as a parsimonious global measure of personality’s association with support, while domain-level analyses were reserved for exploratory models (see Supplementary material). Other factors: family structure, and financial status. The internal consistency (reliability) of the multi-item scales, as measured by Cronbach’s alpha in our sample, was as follows: BFI-10 (*α* = 0.72), PSSS (*α* = 0.91), FHS-SF (*α* = 0.88), EBS-SF (*α* = 0.80), indicating acceptable to good reliability (all in the Supplementary material).

### 2.4 Quality control and Bias mitigation

To ensure data quality, the following measures were implemented: (1) Standardized online training for all investigators; (2) Use of a unified and validated online survey platform; (3) Logical checks and data validation rules within the questionnaire; (4) Monitoring of questionnaire completion time to identify and exclude careless responses.

It is important to acknowledge that the non-probability sampling method, while practical for large-scale national outreach, limits the generalizability of our findings and may introduce selection bias, as participants were recruited through social networks and may not be fully representative of the entire Chinese population.

### 2.5 Statistical analysis

Data were analyzed using SPSS (version 27.0). Descriptive statistics presented counts and percentages for categorical variables and means with standard deviations for continuous variables. Univariate analyses (independent samples t-tests for binary variables; one-way ANOVA for multi-level categorical variables) were conducted to explore associations between individual factors and the support score. Missing data were handled using list wise deletion. A multivariable linear regression analysis was performed to identify factors independently associated with the support score. We used the Enter method, forcing all pre-specified variables from the socio-ecological framework into the model simultaneously, without employing any automated variable selection procedures. The final model included all pre-specified variables from the socio-ecological framework. All variables listed in Section 3.3 were considered as potential independent variables. Categorical variables were dummy-coded (see Supplementary Table 1). The assumptions of linear regression (linearity, homoscedasticity, normality of residuals) were checked and deemed satisfactory. Multicollinearity was assessed using Variance Inflation Factors (VIF), with all VI*F* values below 2.5, indicating no significant multicollinearity.

### 2.6 Ethical considerations

The study protocol was approved by the Institutional Review Board of Ji’nan University, Guangzhou, China (Approval no: JNUKY-2021-018). All participants provided electronic informed consent before participation.

## 3 Results

### 3.1 Participant characteristics

A total of 11,031 valid questionnaires from 23 provinces, 5 autonomous regions, and 4 municipalities in mainland China were analyzed. The sample consisted of 5,033 males (45.6%) and 5,998 females (54.4%). The age distribution was as follows: 12–18 years (9.7%), 19–40 years (48.3%), 41–60 years (31.6%), 61–75 years (7.9%), and over 75 years (2.5%). Detailed sociodemographic characteristics are presented in Table 1.

**Table 1:** A total of 11,031 participants were analyzed in this study.

Categorical variables	*N* (%)	Supporting score, Mean (SD)		*F*-value	*p-*value
Sex					
Male	5,033	73.98	28.518	296.664	0
Female	5,998	82.75	24.959		
Age group					
≤18	1,065	83.43	25.462	21.004	0
19–40	5,332	79.8	26.716		
41–60	3,487	77.18	27.241		
60–75	875	74.65	27.815		
>75	272	73.22	28.485		
Ethnicity					
Han Chinese	10,386	78.98	26.915	12.743	0
Ethnic Minorities	645	75.07	28.035		
Marital status					
Divorced	207	76.37	27.941	25.78	0
Widowed	235	74.31	28.539		
Unmarried	4,363	81.49	26.274		
Married	6,226	77.08	27.243		
Have children					
No	5,062	80.94	26.317	309.426	0.001
Yes	5,969	76.9	27.426		
Highest educational level					
Junior or below	2,566	74.72	28.229	49.945	0
Senior high or specialty education	1978	77.02	27.402		
College degree	1,445	76.8	27.515		
Undergraduate or above	5,042	82.04	25.622		
Religious beliefs					
None	10,709	78.92	26.886	13.844	0
There are	322	73.24	29.97		
Place of residence					
Urban	8,008	79.82	26.626	45.957	0
Rural	3,023	75.92	27.76		
Whether being in debt					
No	6,780	78.32	26.832	4.539	0.033
Yes	4,251	79.44	27.245		
Have you been diagnosed with emotional disorders					
None	10,950	78.75	26.976	0.006	0.937
Yes	81	78.99	29.814		
The frequency of alcohol consumption					
Never	6,581	80.68	25.75	77.689	0
Everyday	197	63.15	30.552		
Every week	1,399	69.07	29.865		
Every month	1,079	77.55	27.396		
<1/month	1775	81.7	26.188		
Whether smoking					
Non-smoker	8,845	82.12	25.001	431.726	0
Smoker	1,399	60.68	30.844		
Ex-smoker	787	72.98	27.562		

### 3.2 Level of support for smoke-free environments

**Figure 1:**
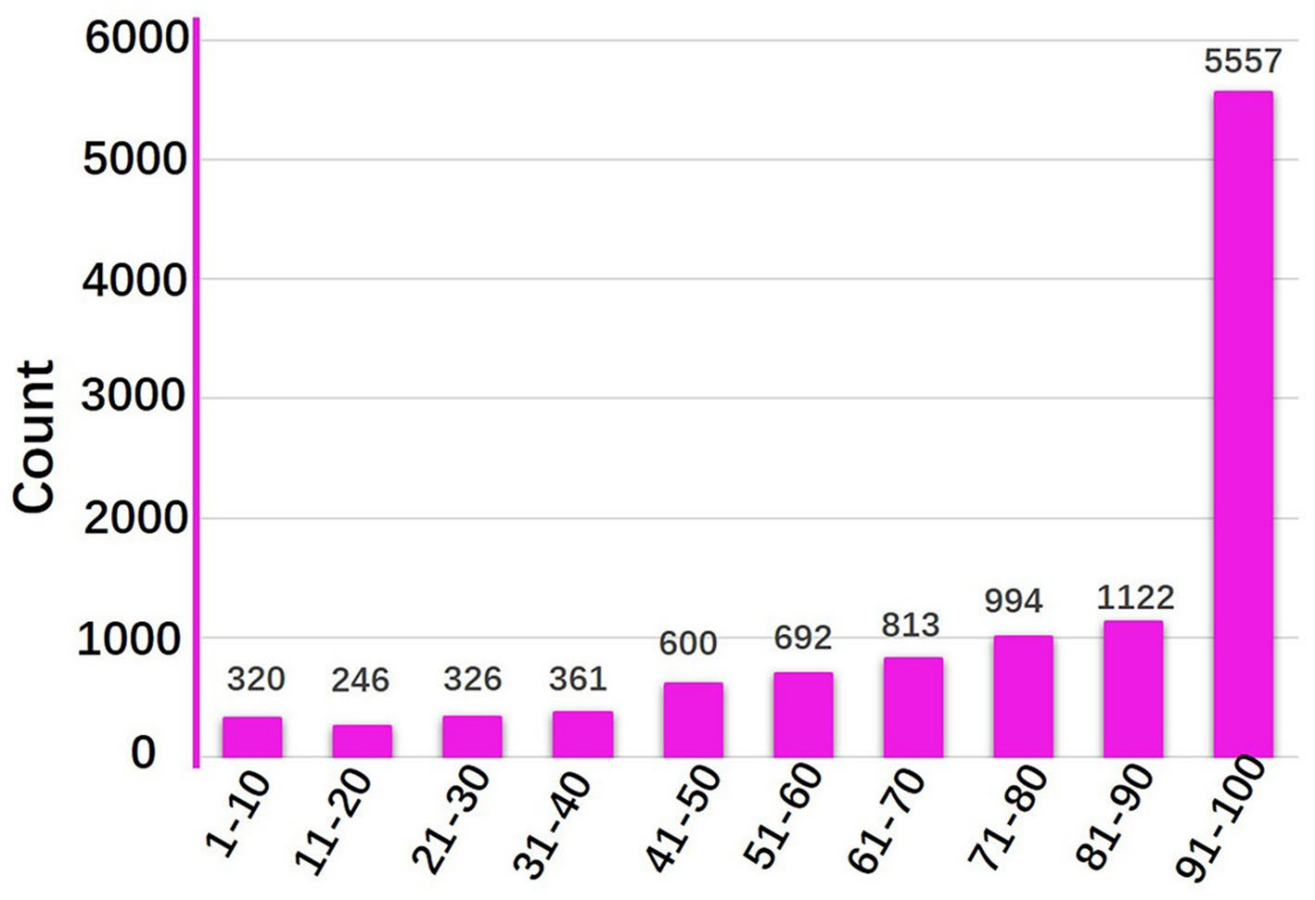
Smoke-free environment support scores.

**Figure 2:**
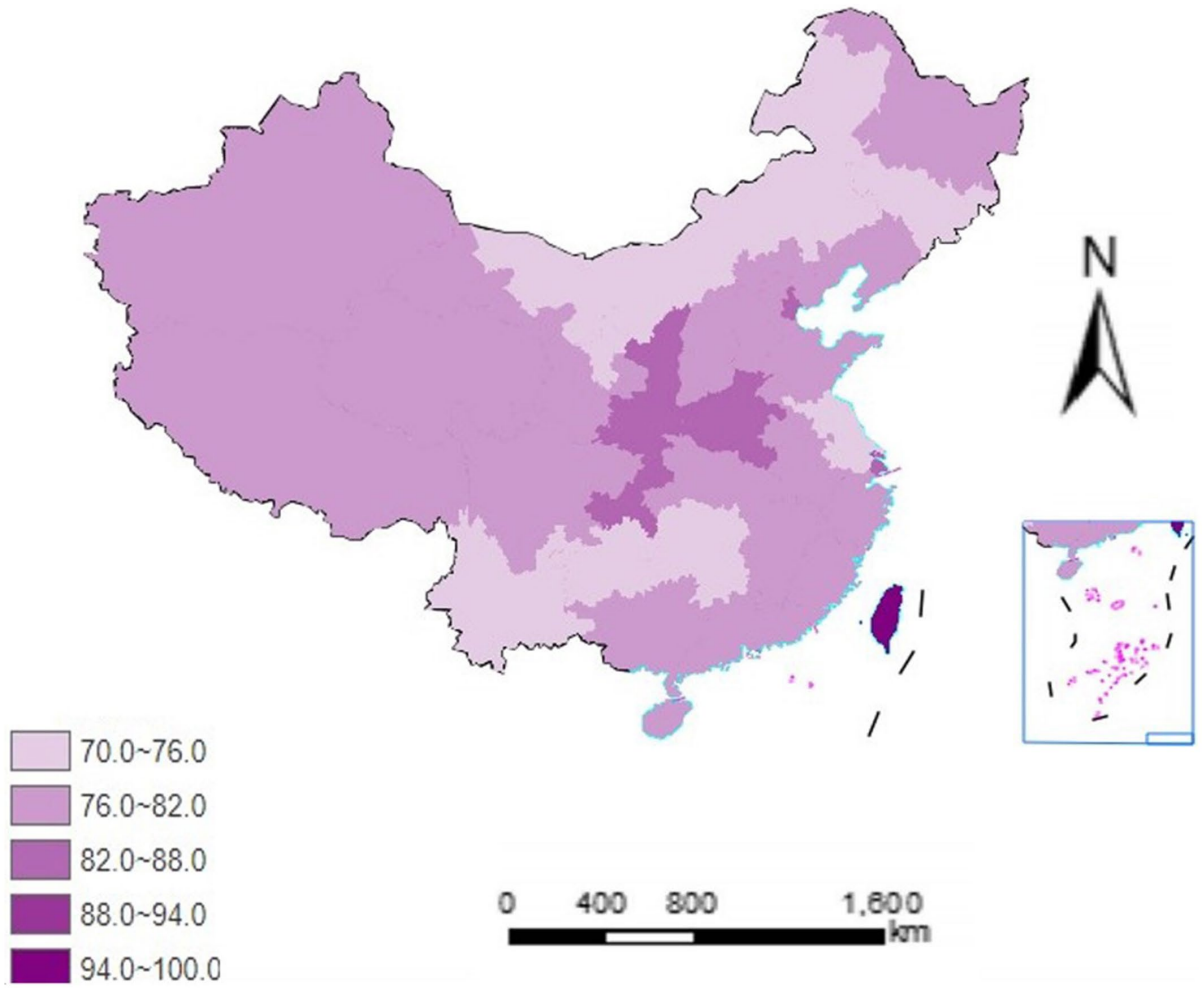
Mean smoke-free environment support scores by province in mainland China.

The mean score for support of smoke-free environments was 78.75 (SD = 26.996), indicating a generally high level of support. More than half of the respondents showed a support score above 50 (Figure 1). Geographic distribution revealed variations across provinces, with central China regions (including Shanxi and Henan) demonstrating relatively higher support scores (Figure 2).

### 3.3 Factors associated with support: multivariable regression analysis

The multiple linear regression model (R^2^ = 0.13) identified several significant correlates of support, which explained 13% of the variance (R^2^ = 0.13) in Table 2.

**Table 2:** Multivariable linear regression analysis of factors associated with smoke-free environment support scores.

Variable	Unstandardized coefficient B		Standardized coefficient	*t*-value	*P-*value	B 95% CI	
	B	SE	Beta			Lower	Upper
Gender (reference: male)							
Female	5.505	0.559	0.101	9.844	0.000	4.409	6.601
Age group (reference: 19–40), y							
41–60	0.428	0.695	0.008	0.616	0.538	−0.933	1.790
61–75	−1.027	1.100	−0.011	−0.933	0.351	−3.183	1.129
>75	−2.504	1.780	−0.015	−1.407	0.159	−5.992	0.985
Ethnicity (reference: Han Chinese)							
Ethnic Minorities	−2.243	1.130	−0.019	−1.984	0.047	−4.458	−0.027
Whether being in debt (reference: no)							
Having debt	0.996	0.532	0.018	1.871	0.061	−0.047	2.040
Whether have children (reference: no)							
Have children	1.554	0.652	0.026	2.384	0.017	0.276	2.833
Whether have emotional disorders (reference: no)							
With emotional disorders	4.810	2.997	0.015	1.605	0.109	−1.064	10.684
Frequency of alcohol consumption (reference: no alcohol consumption)							
Everyday	−5.549	1.901	−0.028	−2.918	0.004	−9.276	−1.822
Every week	−5.275	0.804	−0.067	−6.560	0.000	−6.851	−3.699
Every month	−0.785	0.869	−0.009	−0.904	0.366	−2.488	0.918
BFI-10 scores	−0.559	0.150	−0.038	−3.733	0.000	−0.853	−0.266
EBS-SF scores	−0.328	0.059	−0.057	−5.537	0.000	−0.444	−0.212
Marital status (reference: Unmarried)							
Divorced	−1.482	1.909	−0.008	−0.776	0.438	−5.224	2.260
Widowed	−3.756	1.952	−0.021	−1.924	0.054	−7.582	0.071
Married	−3.375	0.752	−0.060	−4.487	0.000	−4.849	−1.900
Religious beliefs (reference: NO)							
Have	−2.082	1.518	−0.013	−1.371	0.170	−5.058	0.894
FHS-SF scores	0.645	0.052	0.158	12.453	0.000	0.543	0.746
PSSS scores	0.142	0.026	0.068	5.500	0.000	0.092	0.193
Highest educational level (reference: Junior or below)							
Senior high or specialty	0.790	0.878	0.011	0.900	0.368	−0.931	2.510
College degree	1.225	0.953	0.016	1.285	0.199	−0.644	3.093
Undergraduate or above	4.110	0.834	0.076	4.927	0.000	2.475	5.746
Place of residence (reference: Rural)							
Urban	1.151	0.609	0.019	1.891	0.059	−0.042	2.344
Fagerström test for nicotine dependence (FTND) (reference: Mild)							
Moderate	−16.140	1.521	−0.103	−10.609	0.000	−19.122	−13.158
Severe	−21.968	2.909	−0.072	−7.551	0.000	−27.671	−16.265

Sociodemographic and behavioral factors: female gender (*β* = 5.505, 95% CI [4.409, 6.601]), higher education (Undergraduate or above: *β* = 4.110, 95% CI [2.475, 5.746]), and having children (*β* = 1.554, 95% CI [0.276, 2.833]) were associated with stronger support. In contrast, being married (*β* = −3.375, 95% CI [−4.849, −1.900]), frequent drinking (e.g., Weekly: *β* = −5.275, 95% CI [−6.851, −3.699]), and higher tobacco dependence (e.g., Severe: *β* = −21.968, 95% CI [−27.671, −16.265]) were associated with lower support. It is noteworthy that this positive association emerged only after adjusting for confounders in the multivariable model, whereas the univariate analysis showed the opposite trend (see Table 1), highlighting the role of confounding factors.

Psychosocial factors: stronger family health (FHS-SF: *β* = 0.645, 95% CI [0.543, 0.746]) and greater perceived social support (PSSS: *β* = 0.142, 95% CI [0.092, 0.193]) were positive correlates. Conversely, a higher BFI-10 total score (BFI-10: *β* = −0.559, 95% CI [−0.853, −0.266]) and unhealthier eating habits (EBS-SF: *β* = −0.328, 95% CI [−0.444, −0.212]) were negative correlates.

### 3.4 Key insights from subgroup analyses

Stratified analyses revealed that the influence of these factors was not uniform across populations (See Supplementary Tables S3–S5).

*By age*: the strongest predictors varied across life stages, from parental influence in adolescents, to education in young adults, and the pronounced impact of tobacco dependence in middle-aged and older adults (see Table 3).

**Table 3:** Heterogeneity in correlates of support for smoke-free environments by age group.

Variable	19–40 y		41–60 y		61–75 y		>75 y	
	Standardized coefficient *β*	*P*-value	Standardized coefficient *β*	*P*-value	Standardized coefficient *β*	*P*-value	Standardized coefficient *β*	*P*-value
Gender (reference: male)								
Female	0.102	0.000	0.106	0.000	0.075	0.035	0.125	0.059
Ethnicity (reference: Han Chinese)								
Ethnic Minorities	−0.026	0.050	−0.023	0.153	0.024	0.488	−0.067	0.293
Whether being in debt (reference: no)								
Having debt	0.008	0.554	0.001	0.933	0.025	0.450	0.064	0.283
Whether have children (reference: NO)								
Have children	0.017	0.279	0.013	0.414	−0.067	0.056	0.116	0.050
Whether have emotional disorders (reference: no)								
With emotional disorders	0.017	0.178	0.058	0.001	0.008	0.811	0.034	0.580
Frequency of alcohol consumption (reference: no alcohol consumption)								
Everyday	−0.025	0.058	−0.025	0.130	−0.036	0.291	−0.006	0.924
Every week	−0.075	0.000	−0.056	0.002	−0.071	0.042	−0.028	0.643
Every month	−0.010	0.473	0.005	0.786	−0.039	0.250	−0.122	0.041
BFI-10 scores	0.034	0.019	−0.068	0.000	−0.075	0.042	−0.080	0.215
EBS-SF scores	−0.044	0.001	0.014	0.419	0.109	0.016	0.010	0.904
Marital status (reference: Unmarried)								
Divorced	−0.010	0.467	0.000	0.994	−0.063	0.113	0.225	0.003
Widowed	0.011	0.395	−0.021	0.242	−0.083	0.240	0.230	0.148
Married	−0.056	0.001	−0.024	0.303	−0.050	0.493	0.153	0.344
Religious beliefs (reference: no)								
Have	−0.018	0.171	0.011	0.499	0.019	0.563	−0.077	0.216
FHS-SF scores	0.168	0.000	0.170	0.000	0.032	0.405	0.094	0.169
PSSS scores	0.060	0.000	0.072	0.001	0.088	0.041	0.127	0.093
Highest educational level (reference: Junior or below)								
Senior high or specialty	0.032	0.100	−0.036	0.056	0.060	0.085	0.164	0.008
College degree	0.030	0.142	−0.014	0.476	0.052	0.144	0.107	0.068
Undergraduate or above	0.093	0.000	0.051	0.014	0.018	0.626	−0.134	0.040
Place of residence (reference: Rural)								
Urban	0.000	0.980	0.031	0.054	−0.007	0.826	0.025	0.682
Fagerström Test for Nicotine Dependence (FTND) (reference: Mild)								
Moderate	−0.088	0.000	−0.121	0.000	−0.090	0.007	−0.051	0.418
Severe	−0.077	0.000	−0.078	0.000	−0.070	0.036	−0.084	0.167

*By gender*: the positive association of having children and family health was stronger among females, while the negative impact of tobacco dependence and drinking was more pronounced among males (see Table 4).

**Table 4:** Heterogeneity in the correlates of support for smoke-free environments by gender.

Variable	Male				Female			
	Standardized coefficient *β*	*P*-value	B 95% CI		Standardized coefficient *β*	*P*-value	B 95%CI	
			Lower	Upper			Lower	Upper
Age group (reference: 19–40), y								
41–60	0.013	0.458	−1.314	2.916	−0.002	0.890	−1.889	1.640
61–75	−0.008	0.625	−4.029	2.421	−0.025	0.120	−5.130	0.589
>75	−0.022	0.160	−8.742	1.446	−0.025	0.097	−8.683	0.722
Ethnicity (reference: Han Chinese)								
Ethnic Minorities	−0.006	0.683	−4.387	2.874	−0.032	0.015	−6.177	−0.658
Whether have children (reference: no)								
Have children	0.005	0.758	−1.662	2.283	0.051	0.001	1.072	4.415
Whether have emotional disorders (reference: no)								
With emotional disorders	0.009	0.516	−6.160	12.262	0.018	0.165	−2.200	12.888
Frequency of alcohol consumption (reference: no alcohol consumption)								
Everyday	−0.032	0.026	−9.426	−0.614	−0.026	0.046	−17.247	−0.151
Every week	−0.080	0.000	−7.244	−3.349	−0.047	0.000	−8.850	−2.555
Every month	−0.014	0.352	−3.329	1.185	−0.002	0.861	−2.989	2.499
BFI-10 scores	0.027	0.094	−0.030	0.387	0.034	0.022	0.028	0.359
EBS-SF scores	−0.049	0.001	−0.480	−0.116	−0.067	0.000	−0.509	−0.210
Marital status (reference: Unmarried)								
Divorced	−0.005	0.713	−7.596	5.198	−0.017	0.226	−7.352	1.738
Widowed	−0.026	0.098	−12.445	1.055	−0.023	0.155	−7.943	1.264
Married	−0.038	0.052	−4.565	0.018	−0.085	0.000	−6.258	−2.422
Religious beliefs (reference: no)								
Have	−0.012	0.406	−7.335	2.971	−0.015	0.263	−5.609	1.529
FHS-SF scores	0.138	0.000	0.423	0.742	0.183	0.000	0.574	0.836
PSSS scores	0.088	0.000	0.110	0.266	0.053	0.002	0.040	0.173
Highest educational level (reference: Junior or below)								
Senior high or specialty	0.009	0.634	−1.974	3.242	0.013	0.429	−1.366	3.215
College degree	0.016	0.375	−1.586	4.206	0.014	0.424	−1.441	3.425
Undergraduate or above	0.085	0.000	2.395	7.445	0.065	0.003	1.109	5.408
Place of residence (reference: Rural)								
Urban	0.019	0.205	−0.669	3.116	0.021	0.134	−0.360	2.684
Debt or not (reference: no)								
Have debt	−0.005	0.702	−1.968	1.326	0.038	0.004	0.611	3.277
Fagerström test for nicotine dependence (FTND) (reference: Mild)								
Moderate	−0.138	0.000	−19.559	−12.965	−0.036	0.007	−28.185	−4.521
Severe	−0.108	0.000	−31.207	−18.393	0.006	0.666	−13.953	21.826

*By smoking status*: the negative association of tobacco dependence was most pronounced among current smokers. In contrast, former and never-smokers’ support was more strongly influenced by psychosocial factors like family health and social support (see Table 5).

**Table 5:** Heterogeneity in the correlates of support for smoke-free environments by smoking status.

Variable	Smoking and quit smoking				Never smoking			
	Standardized coefficient *β*	*P*-value	B 95% CI		Standardized coefficient *β*	*P*-value	B 95%CI	
			Lower	Upper			Lower	Upper
Gender (reference: male)								
Female	0.054	0.012	1.211	9.906	0.055	0.000	1.721	4.086
Age group (reference: 19–40), y								
41–60	0.028	0.299	−1.514	4.917	0.007	0.638	−1.122	1.830
61–75	0.007	0.804	−4.041	5.216	−0.005	0.685	−2.949	1.939
>75	−0.012	0.635	−8.684	5.302	−0.008	0.508	−5.450	2.697
Ethnicity (reference: Han Chinese)								
Ethnic Minorities	−0.007	0.745	−6.235	4.462	−0.021	0.058	−4.695	0.074
Whether have children (reference: no)								
Have children	0.016	0.496	−1.894	3.912	0.033	0.011	0.415	3.221
Whether have emotional disorders (reference: no)								
With emotional disorders	0.024	0.258	−6.023	22.462	0.012	0.279	−2.824	9.803
Frequency of alcohol consumption (reference: No alcohol consumption)								
Everyday	−0.037	0.097	−9.779	0.810	0.000	0.965	−6.818	6.518
Every week	−0.064	0.007	−6.837	−1.090	−0.014	0.222	−3.484	0.810
Every month	0.003	0.898	−3.411	3.890	0.003	0.771	−1.654	2.231
BFI-10 scores	0.003	0.886	−0.312	0.361	0.035	0.004	0.064	0.340
EBS-SF scores	−0.047	0.040	−0.604	−0.014	−0.059	0.000	−0.437	−0.190
Marital status (reference: Unmarried)								
Divorced	0.004	0.868	−7.478	8.860	−0.001	0.915	−4.427	3.971
Widowed	0.000	1.000	−8.338	8.341	−0.018	0.142	−7.523	1.078
Married	0.032	0.263	−1.711	6.267	−0.074	0.000	−5.308	−2.189
Religious beliefs (reference: no)								
Have	−0.017	0.438	−9.896	4.290	−0.012	0.266	−5.043	1.391
FHS-SF scores	0.071	0.014	0.064	0.565	0.194	0.000	0.629	0.846
PSSS scores	0.112	0.000	0.129	0.380	0.053	0.000	0.049	0.158
Highest educational level (reference: Junior or below)								
Senior high or specialty	−0.027	0.281	−5.575	1.621	0.023	0.099	−0.311	3.592
College degree	0.024	0.360	−2.162	5.954	0.015	0.313	−1.014	3.161
Undergraduate or above	0.028	0.332	−1.895	5.610	0.074	0.000	1.897	5.521
Place of residence (reference: Rural)								
Urban	0.001	0.975	−2.882	2.978	0.023	0.046	0.021	2.576
Debt or not (reference: no)								
Have debt	0.015	0.491	−1.701	3.544	0.027	0.016	0.260	2.481
French Tobacco Dependence Scale (FTND) (reference: Mild)								
Moderate	−0.129	0.000	−14.760	−7.557				
Severe	−0.101	0.000	−22.970	−9.508				

## 4 Discussion

Guided by the socio-ecological model, this study examines the multifaceted determinants of public support for smoke-free environments in China. Our findings not only confirm the roles of established sociodemographic factors but also uncover novel psychosocial pathways, offering a nuanced evidence base for targeted policy interventions.

### 4.1 Key determinants and novel pathways

The generally high level of public support (mean score: 78.75) indicates a fertile ground for advancing smoke-free policies in China. Consistent with global evidence (3, 5–8), being female and having a higher educational attainment were significant positive correlates. The stronger support among women may be linked to their heightened health awareness and role as family health guardians, particularly regarding secondhand smoke exposure (10–14).

More importantly, our study extends beyond demographics to reveal critical psychosocial mechanisms. The independent positive associations of family health (FHS-SF) and perceived social support (PSSS) with policy support suggest that interventions strengthening family dynamics and social networks could be highly effective. This finding bridges the gap between literature on social support in smoking cessation (15–20) and public policy endorsement, proposing a novel avenue for mobilizing community-level support. This finding addresses a key gap in the literature by demonstrating that psychosocial determinants, independent of demographics, play a crucial role in shaping policy support. Furthermore, the observed negative association between the global BFI-10 score and policy support suggests that broader personality dispositions may also influence an individual’s receptiveness to public health regulations. While the use of a total score limits specificity, this finding is consistent with studies that have successfully used this concise instrument to link personality to health-related behaviors (9). It resonates with research linking certain personality profiles (e.g., those lower in agreeableness or conscientiousness) to a greater propensity for health-risk behaviors, including smoking (21, 22). This indicates that individuals with such inherent tendencies may be less supportive of policies they perceive as restricting personal freedom.

### 4.2 Complex behavioral syndromes and subgroup heterogeneity

The clustering of lower support with frequent drinking and unhealthy eating patterns points to a underlying health-risk behavior syndrome. This synergy, supported by existing research on co-occurring risky behaviors (23–33), advocates for integrated public health initiatives that address these behaviors collectively rather than in isolation. Our stratified analyses further provide a blueprint for precision public health. The dominant influence of nicotine dependence among current smokers underscores that policy acceptance in this group is contingent on effective cessation support. Conversely, never-smokers are more responsive to educational and family-protection messaging. These patterns, along with notable gender differences, affirm that tobacco control strategies must be subgroup-specific to resonate with distinct motivations and barriers (34–36).

### 4.3 Limitations

The interpretations of our findings should be considered in light of several limitations. First, the cross-sectional design precludes causal inference. Second, the non-probability sampling method may limit the generalizability of our results. Third, the model’s R^2^ of 0.13 indicates that a substantial portion of the variance remains unexplained, highlighting the complexity of this phenomenon and the influence of unmeasured factors. Additionally, the use of a total score for the BFI-10, while parsimonious, limits our ability to identify which specific personality traits underlie the observed association with support.

## 5 Conclusion and implications

In conclusion, this study demonstrates that support for smoke-free environments in China is a complex phenomenon shaped by a web of socio-ecological factors. To effectively cultivate this support, a one-size-fits-all approach is insufficient. We recommend: (1) Developing targeted communication campaigns that leverage family-centric messages for women and health-consequence-focused messaging for men. (2) Implementing integrated health programs that address smoking, drinking, and diet as interconnected behaviors. (3) Prioritizing smoking cessation support as a dual-benefit strategy that simultaneously improves individual health and builds policy acceptance among smokers.

Future research should employ longitudinal designs to establish causality and explore the mechanisms behind complex associations, such as the lower support among married individuals. By adopting such a multi-level and tailored strategy, China can more effectively harness public will to achieve its smoke-free goals under the WHO FCTC framework (4).

## Data Availability

The datasets presented in this study can be found in online repositories. The names of the repository/repositories and accession number(s) can be found in the article/Supplementary material.
