# Supplementary material for: Public attitudes about the support for the establishment of smoke-free environment
Source: Front Public Health. 2025 Dec 16;13:1646224. doi: 10.3389/fpubh.2025.1646224 (PMC12748262; doi:10.3389/fpubh.2025.1646224)
Supplement: Supplementary file 1 [file Data_Sheet_1.docx]

**Supplemental Material**

**S Methods.**

**S References.**

**S Table 1. The dummy coding of categorical variables**

**S Table 2. The statistical description of metric variables**

**S Table 3 Family health and their entry scores for the four dimensions**

**S Table 4 Perceived social support scale and their entry scores for the four dimensions**

**S Table 5 Mean and SD for Big five personality domains and raw aspect scores**

**S Figure 1. Figure 1 Smoking harms nearly every organ of the body**

**S Figure 2. The Ecological Model of Health Behavior and the research hypotheses**

**S Figure 3. Flowchart of participant enrollment**

**This supplementary material has been provided by the authors to give readers additional**

**information about their work.**

**S Methods.**

**Research Instruments**

**A visual analogue scale (VAS)** was applied to evaluate willingness to support, using a scale from 0 to 100，A higher score indicates greater willingness to support. Respondents chose the score according to their willingness.

**Family Health Scale-Short Form，FHS-SF：**The scale was compiled by Crandall and Weiss laxer **^1^**and translated into Chinese by members of the China family newspaper research center according to the standard translation process of the scale. **FHS-SF** is composed of 2-3 items with large weight and factor load from the four dimensions of family social and emotional health process, family healthy lifestyle, family health resources, and family external social support in the family health scale long form (**FHS-LF**), with a total of 10 items. Each item is scored by Likert 5-level scoring method, of which questions 6, 9, and 10 are scored in reverse, The higher the score, the higher the family health level. The Cronbach α coefficient for the **FHS-SF** scale was 0.849, indicating that the scale has good internal consistency reliability.

**Perceived social support scale，PSSS：**The scale was developed by zimet GD, etc. **^2^**

In this part, we used the Chinese version of the scale translated by Jiang Qianjin, etc. The scale is divided into three dimensions: family support (4 items), friend support (4 items) and other support (relatives, neighbors and colleagues) (4 items), with a total of 12 items. Each item uses Likert 7-level scoring method, and the score ranges from extremely disagree (0 points) to extremely agree (6 points). The scores of all items in each dimension is the score of this dimension, and the total score reflects the total degree of social support that individuals feel. **The total score of the scale between 0-24 was in the low support state; The total score between 25-48 is intermediate support status; The higher the total score, the higher the individual's perception of social support.** The Cronbach α coefficient for the **PSSS** scale was 0.871, indicating that the scale has good internal consistency reliability.

**New General Self-Efficacy Scale，NGSES：**The scale was adapted based on the general self-efficacy scale (GSSE) to measure people's self-efficacy by Chen G. etc.**^3^** There are 8 items in the scale, all of which are positive scoring. Each item adopts Likert 5-level scoring method, and the score ranges from very disagree (1 point) to very agree (5 points). The higher the standard score, the better the self-efficacy. The Cronbach α coefficient for the **NGSES** score was 0.794, indicating that the scale has good internal consistency reliability.

**Euro quality of life five-dimension questionnaire，EQ-5D-5L：** Participants’ quality of life and self-perceived health were assessed with the EQ-5D-5L across 5 dimensions: mobility, self-care, usual activities, pain and discomfort, and anxiety and depression**^4^**. Scores ranged from 1 to 5 on a Likert-type scale, with a maximum total score of 25 and higher scores indicating poorer quality of life. In this study, the Cronbach α coefficient for the EQ-5D-5L scale was 0.811.

**The Short-Form of the Eating Behavior Scale (EBS-SF):** the short-form of the Eating Behavior Scale was applied in this study to assess the eating behavior of residents**^5^** . The EBS-SF was scored on a 4-point Likert scale indicating the residents’ degree of agreement (1 = strongly disagree, 2 = somewhat disagree, 3 = somewhat agree, and 4 = strongly agree), and seven items were summed to obtain scores between 7 and 28, with higher scores indicating worse eating behavior. The Cronbach α coefficient for the EBS-SF score in the present study was 0.871, showing that it had good internal consistency.

**Media use score:** Media use was a scale developed for this study to assess individuals’ media consumption behaviors, based on relevant literature.27,28 The scale included 6 items that correspond to 6 different types of media use behavior: social communication, self-presentation, social action (such as advocacy and promoting justice), leisure and entertainment, information acquisition through media, and commercial transactions. Each item was scored using a Likert-type 5-point scale, ranging from 1 (never used) to 5 (always used). The total score ranges from 6 to 30, with higher scores indicating more frequent media use. In this study, the Cronbach α coefficient for the media use scale was 0.872.

**The 10-item Big Five Inventory，BFI-10:** This scale was developed by Courtois R et al, and is based on the Big Five personality model **^6^**. It is divided into five dimensions: neuroticism (2 items), extraversion (2 items), openness (2 items), agreeableness (2 items), and sense of responsibility (2 items), with a total of 10 items. Each item is scored using the Likert 5-point scoring method, ranging from strongly disagree (1 point) to strongly agree (5 points). Items 1, 3, 5, 7, and 9 are reverse scores, and the scores of all items in each dimension are added together to form the Likert 5-point scale. The score of this dimension, the higher the corresponding dimension score, the more suitable the subject is for this personality dimension.

The Cronbach α coefficient for the BFI-10 scale was 0.716，indicating that the scale has good internal consistency reliability.

**French Tobacco Dependence Scale, (FTND):** is a widely used tool to assess the level of nicotine dependence in smokers. The scale including the following 6 questions: 1. How soon after you wake up do you smoke your first cigarette? 2. Do you find it difficult to refrain from smoking in places where it is forbidden, e.g. in church, at the library, in the cinema? 3. Which cigarette would you hate most to give up? 4. How many cigarettes per day do you smoke? 5. Do you smoke more frequently during the first hours after waking than the rest of the day? 6. Do you smoke when you are so ill that you are in bed most of the day? The degree of tobacco dependence can be divided into the following categories: Mild dependence: 1~3 points; Moderate dependence: 4~6 points; Severe dependence: ≥7 points.**^7^**

**The Ecological Model:** The Ecological Model was widely adopted to analyze the influencing factors of specific health behavior which including personal characteristics, individual behaviors, interpersonal networks, community, and public policy. In the present study, we divided the data into five level. As we have mentioned in the content above.

1. Crandall A, Weiss-Laxer NS, Broadbent E, et al. The Family Health Scale: Reliability and Validity of a Short- and Long-Form. *Front Public Health.* 2020;8:587125.

2. Zimet GD, Powell SS, Farley GK, Werkman S, Berkoff KA. Psychometric characteristics of the Multidimensional Scale of Perceived Social Support. *J Pers Assess.* 1990;55(3-4):610-617.

3. Chen G, Gully SM, Eden D. Validation of a New General Self-Efficacy Scale. *Organizational Research Methods.* 2001;4(1):62-83.

4. Herdman M, Gudex C, Lloyd A, et al. Development and preliminary testing of the new five-level version of EQ-5D (EQ-5D-5L). *Qual Life Res.* 2011;20(10):1727-1736.

5. Mei D, Deng Y, Li Q, et al. Current Status and Influencing Factors of Eating Behavior in Residents at the Age of 18~60: A Cross-Sectional Study in China. *Nutrients.* 2022;14(13).

6. Courtois R, Petot JM, Plaisant O, et al. [Validation of the French version of the 10-item Big Five Inventory]. *Encephale.* 2020;46(6):455-462.

7. Berlin I, Singleton EG, Heishman SJ. Validity of the 12-item French version of the Tobacco Craving Questionnaire in treatment-seeking smokers. *Nicotine Tob Res.* 2010;12(5):500-507.

**S Table 1 The dummy coding of categorical variables**

| Categorical variables | Dummy coding |
| --- | --- |
| Sex  Male Female | 1  2 |
| Age group  ≤18  19-40  41-60  60-75  ＞75 | 1  2  3  4  5 |
| BMI  ＜18  18-24  ＞24  ＞28 | 1  2  3  4 |
| Ethnicity  Han Chinese  Ethnic Minorities | 1  2 |
| Marital status  Divorced  widowed  unmarried  married | 1  2  3  4 |
| Have children  No  Yes | 1  2 |
| Highest educational level  Junior or below  Senior high or specialty education  College degree  Undergraduate or above | 1  2  3  4 |
| Political Status  Masses  Members  Party members  Others | 1  2  3  4 |
| Religious beliefs  None  There are | 1  2 |
| Place of residence  Urban  Rural | 1  2 |
| Type of resident visa  Urban  Rural | 1  2 |
| What type of subsidy do you accept  Unclear  Social subsidies  No subsidies received  Government subsidies | 1  2  3  4 |
| Number of properties owned  No  One  Two  More the three | 1  2  3  4 |
| Debt or not  No  Yes | 1  2 |
| Current monthly average income  ＜1500  1500-6000  6000-12000  ＞12000 | 1  2  3  4 |
| Current occupational status  Retired  No fixed occupation  Student  On the job | 1  2  3  4 |
| Type of occupation engaged in  Medical personnel  Party and government officials  Professional technical personnel  Others | 1  2  3  4 |
| Medical insurance  Public medical insurance  Resident medical insurance  Commercial medical insurance  Employee medical insurance  Self-funded | 1  2  3  4  5 |
| Diagnosed chronic diseases  None  There are | 1  2 |
| Have you been diagnosed with emotional disorders  None Yes | 1  2 |
| Do you have any disabilities  None  Yes | 1  2 |
| Have you ever drunk alcohol in the past 12 months?  Within the past 30 days  In the past 30 days  Never  In the past 12 months | 1  2  3  4 |
| The frequency of alcohol consumption  Never  Everyday  Every week  Every month  ＜1/month | 1  2  3  4  5 |
| Age of first drinking  Never  ≤10  11-18  ≥19 | 1  2  3  4 |
| Smoking or not  Never smoked  Smoking  Have quit smoking | 1  2  3 |
| Age of first smoking a cigarette  Never  ≤10  11-18  ≥19 | 1  2  3  4 |
| The number of cigarettes smoked per day  None  ≤10  11-20  21-30  ≥31 | 1  2  3  4  5 |
| First cigarette after waking up in the morning  None  ＞60min  31-60min  6-30min  ≤5min | 1  2  3  4  5 |
| Is it difficult to control smoking in non-smoking areas  None  No  Yes | 1  2  3 |
| The cigarette you least want to give up  None  The first one in the morning  Other time | 1  2  3 |
| Smoke more in the first hour of waking up in the morning  None  No  Yes | 1  2  3 |
| Sick in bed still smoking  None  No  Yes | 1  2  3 |

**S Table 2. The statistical description of metric variables.**

| **Metric Variables** | **M ± SD** |
| --- | --- |
| **BFI-10 scores** | **33.02±4.329** |
| **PSSS scores** | **48.22±13.027** |
| **FHS-SF scores** | **37.99±6.640** |
| **Media use score** | **19.34±4.963** |
| **EQ-5D-5L scores** | **5.84±1.824** |
| **EBS-SF scores** | **16.45±4.653** |

**S Table 3 Family health and their entry scores for the four** **dimensions**

| **FHS-SF（Family Health）** | **Mean±SD** | **Male (Mean±SD)** | **Female**  **(Mean±SD)** | **P Value** |
| --- | --- | --- | --- | --- |
| **Family social/emotional health processes** | **11.89±1.605** | **11.7580±2.63692** | **11.9995±2.47930** | **0.000** |
| We support each other | **3.96±0.915** | **3.91±0.939** | **4.00±0.891** | **0.000** |
| I feel safe in my family relationships | **3.93±0.953** | **3.90±0.965** | **3.95±0.942** | **0.003** |
| We stay hopeful even in difficulty time | **4.00±0.897** | **3.95±0.933** | **4.05±0.864** | **0.000** |
| **Family healthy lifestyle** | **8.00±1.298** | **7.9001±1.78520** | **8.0834±1.68547** | **0.000** |
| We help each other in seeking health care services when needed (such as making doctor’s appointments) | **4.03±0.913** | **3.97±0.942** | **4.08±0.885** | **0.000** |
| We help each other make healthy changes | **3.97±0.920** | **3.93±0.943** | **4.01±0.899** | **0.000** |
| **Family health resources** | **10.48±2.151** | **10.3326±3.09036** | **10.5979±3.03094** | **0.000** |
| We do not trust doctors and other health professionals | **3.72±1.220** | **3.62±1.228** | **3.79±1.207** | **0.000** |
| My family did not have enough money at the end of the month after bills were paid | **3.23±1.211** | **3.23±1.207** | **3.23±1.215** | **0.821** |
| My family did not have adequate housing | **3.53±1.226** | **3.48±1.225** | **3.57±1.224** | **0.000** |
| **Family external social supports** | **7.63±1.308** | **7.5245±1.70436** | **7.7124±1.61592** | **0.000** |
| We have people outside of our family we can turn to when we have problems at school or work | **3.77±0.886** | **3.72±0.900** | **3.80±0.872** | **0.000** |
| If we needed financial help, we have people outside of our family we could turn to for a loan (e.g., for ¥1000) | **3.86±0.962** | **3.80±0.981** | **3.91±0.943** | **0.000** |

**S Table 4 Perceived social support scale and their entry scores for the four dimensions**

| **Perceived social support scale，PSSS** | **Mean±SD** | **Mean±SD** | | **P Value** |
| --- | --- | --- | --- | --- |
|  |  | **Male** | **Female** |  |
| **Family support** | **16.21±2.676** | **15.9825±4.87414** | **16.3989±4.66424** | **0.000** |
| My family tries to help me. | **3.97±1.352** | **3.94±1.372** | **3.99±1.335** | **0.087** |
| I get emotional help and support from my family. | **4.14±1.349** | **4.07±1.379** | **4.20±1.322** | **0.000** |
| I can talk about my problems with my family. | **4.05±1.343** | **3.97±1.375** | **4.11±1.312** | **0.000** |
| My family is willing to help me make decisions. | **4.05±1.323** | **3.99±1.355** | **4.11±1.294** | **0.000** |
| **Friend support** | **16.00±2.551** | **15.7129±4.79641** | **16.2377±4.52818** | **0.000** |
| My friends really try to help me. | **3.99±1.286** | **3.93±1.326** | **4.04±1.249** | **0.000** |
| I can count on my friends when things go wrong. | **3.91±1.318** | **3.87±1.342** | **3.87±1.342** | **0.004** |
| I have friends with whom I share joys and sorrows. | **4.07±1.256** | **3.97±1.297** | **4.16±1.215** | **0.000** |
| I can talk about my problems with my friends. | **4.03±1.270** | **3.95±1.308** | **4.09±1.233** | **0.000** |
| **Other support (relatives, neighbors and colleagues)** | **16.01±2.622** | **15.6265±4.74623** | **16.3414±4.40729** | **0.000** |
| There is a special person around when I am in need. | **3.98±1.305** | **3.88±1.355** | **4.07±1.255** | **0.000** |
| There is a special person I share joys and sorrows with. | **3.96±1.325** | **3.85±1.376** | **4.05±1.274** | **0.000** |
| I have a special person who Is a source of comfort. | **4.02±1.321** | **3.96±1.315** | **4.14±1.240** | **0.000** |
| There is a special person who cares about feelings. | **4.06±1.278** | **3.93±1.367** | **4.09±1.276** | **0.000** |

**S Table 5 Mean and SD for Big five personality domains and raw aspect scores**

| **The 10-item Big Five Inventory，BFI-10** | **Mean±SD** | **Mean±SD** | | **P Value** |
| --- | --- | --- | --- | --- |
|  |  | **Male** | **Female** |  |
| **Extraversion (item1 and item6),** | **6.29± 1.549** | **6.4590±1.51514** | **6.4718±1.54660** | **0.661** |
| **Agreeableness (item 2 and item 7),** | **7.00±1.458** | **6.9062±1.48721** | **7.0774±1.49414** | **0.000** |
| **Consciousness (item 3 and item 8),** | **6.84± 1.512** | **6.7407±1.54687** | **6.8861±1.61705** | **0.000** |
| **Neuroticism (item 4 and item 9),** | **6.26± 1.478** | **6.4250±1.44823** | **6.1260±1.52675** | **0.000** |
| **Openness (item 5and item 10),** | **6.47±1.496** | **6.3890±1.49005** | **6.5450±1.57442** | **0.000** |

**
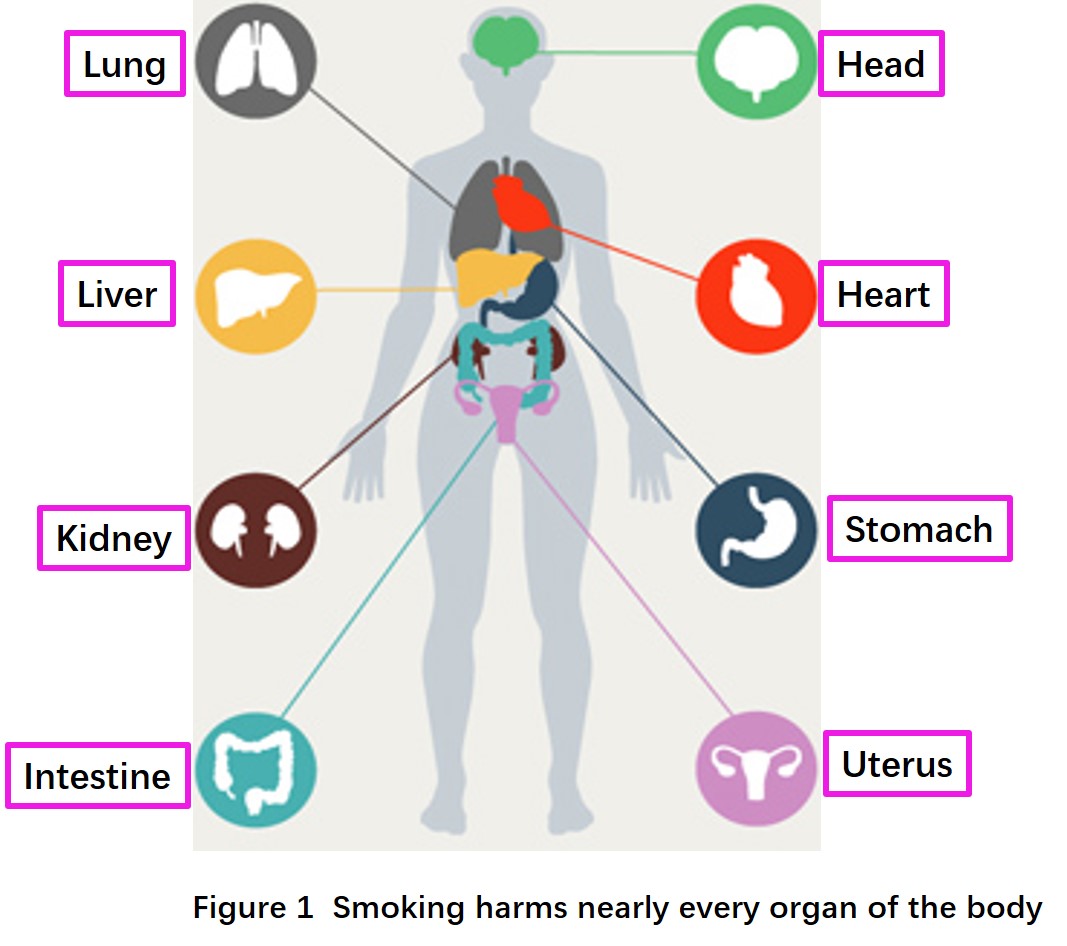
**

**S Figure 1 Smoking harms nearly every organ of the body**

**
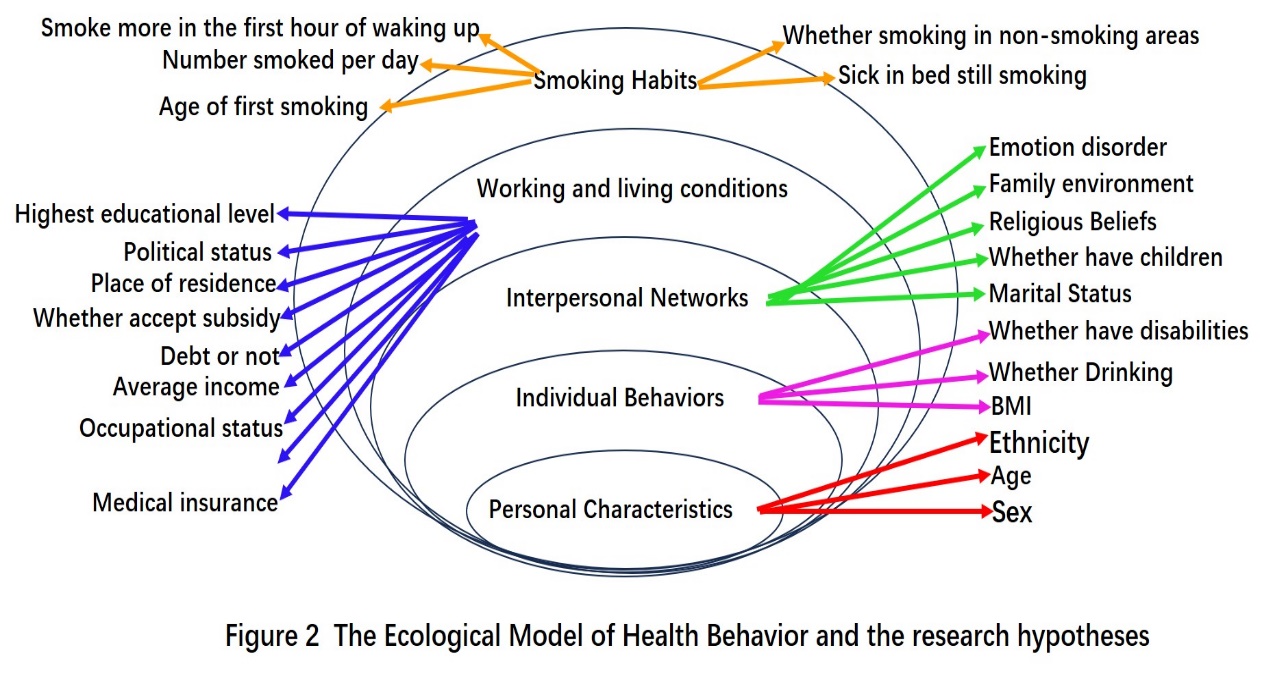
**

**S Figure 2 The Ecological Model of Health Behavior and the research hypotheses**

**
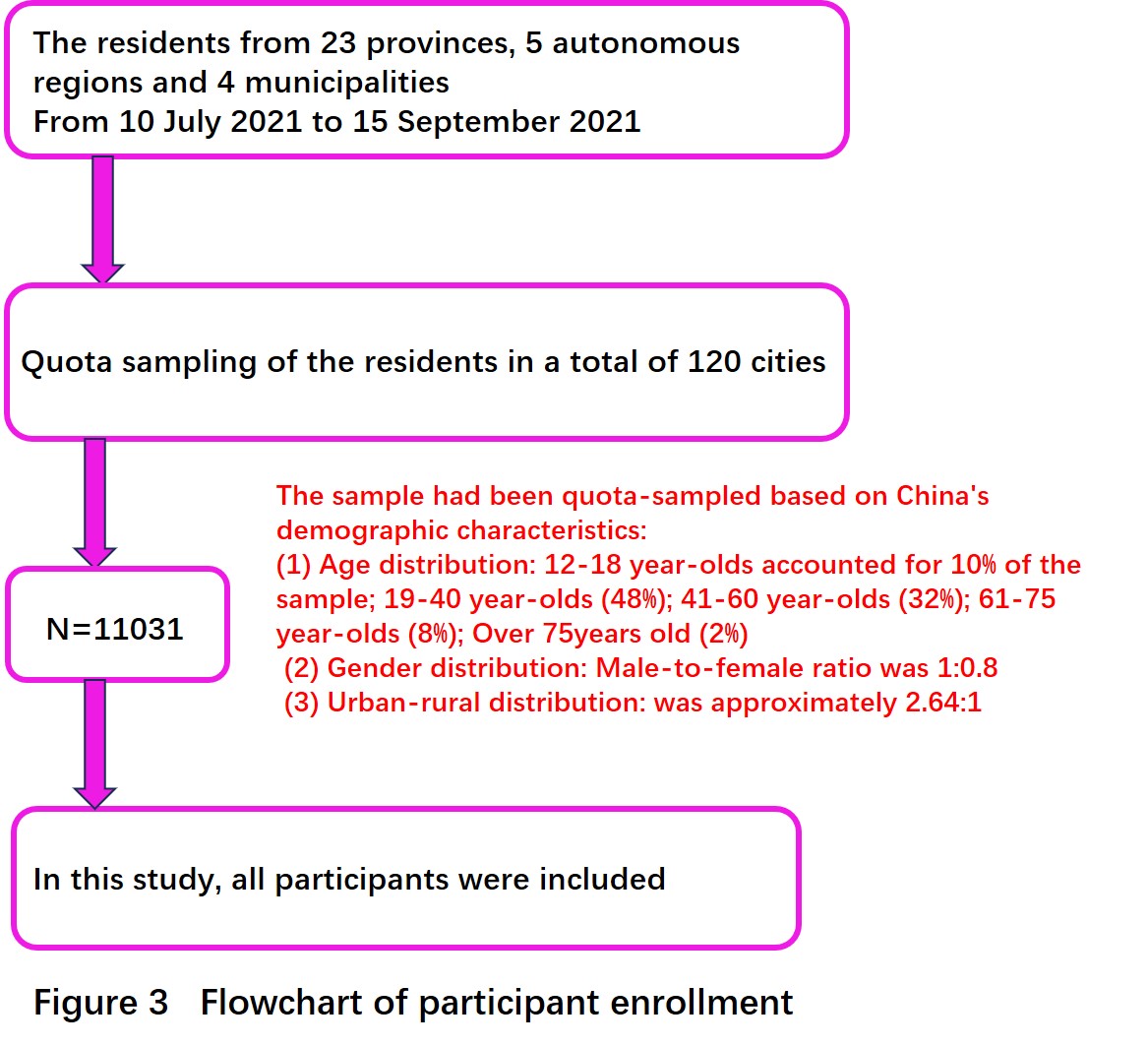
S Figure 3 Flowchart of participant enrollment**
